# Investigating autonomic nervous system dysfunction among patients with post-COVID condition and prolonged cardiovascular symptoms

**DOI:** 10.3389/fmed.2023.1216452

**Published:** 2023-10-09

**Authors:** Fernanda Stábile da Silva, Lívia Pimenta Bonifácio, Fernando Bellissimo-Rodrigues, Luis Fernando Joaquim, Daniel Penteado Martins Dias, Minna Moreira Dias Romano, André Schmidt, Júlio César Crescêncio, Tereza C. Buzinari, Rubens Fazan, Helio Cesar Salgado

**Affiliations:** ^1^Department of Physiology, Ribeirão Preto Medical School, University of São Paulo, Ribeirão Preto, Brazil; ^2^Department of Social Medicine, Ribeirão Preto Medical School, University of São Paulo, Ribeirão Preto, Brazil; ^3^Centro Universitário Barão de Mauá, Ribeirão Preto, Brazil; ^4^Department of Internal Medicine, Ribeirão Preto Medical School, University of São Paulo, Ribeirão Preto, Brazil

**Keywords:** heart rate variability, blood pressure variability, COVID-19, post-COVID-19 syndrome, head-up tilt test

## Abstract

Heart Rate Variability (HRV) and arterial pressure (AP) variability and their responses to head-up tilt test (HUTT) were investigated in Post-COVID-19 syndrome (PCS) patients reporting tachycardia and/or postural hypotension. Besides tachycardia, PCS patients also showed attenuation of the following HRV parameters: RMSSD [square root of the mean of the sum of the squares of differences between adjacent normal-to-normal (NN) intervals] from statistical measures; the power of RR (beat-to-beat interval) spectra at HF (high frequency) from the linear method spectral analysis; occurrence of 2UV (two unlike variation) pattern of RR from the nonlinear method symbolic analysis; and the new family of statistics named sample entropy, when compared to control subjects. Basal AP and LF (low frequency) power of systolic AP were similar between PCS patients and control subjects, while 0 V (zero variation) patterns of AP from the nonlinear method symbolic analysis were exacerbated in PCS patients. Despite tachycardia and a decrease in RMSSD, no parameter of HRV changed during HUTT in PCS patients compared to control subjects. PCS patients reassessed after 6 months showed higher HF power of RR spectra and a higher percentage of 2UV pattern of RR. Moreover, the reassessed PCS patients showed a lower occurrence of 0 V patterns of AP, while the HUTT elicited HR (heart rate) and AP responses identical to control subjects. The HRV and AP variability suggest an autonomic dysfunction with sympathetic predominance in PCS patients. In contrast, the lack of responses of HRV and AP variability indices during HUTT indicates a marked impairment of autonomic control. Of note, the reassessment of PCS patients showed that the noxious effect of COVID-19 on autonomic control tended to fade over time.

## Introduction

Heart rate variability (HRV) has been thoroughly investigated as a way to evaluate changes in the neural control of the heart in patients with post-COVID-19 syndrome (PCS) (1–3). However, it is worth mentioning that most of the studies that have evaluated HRV in COVID-19 focused on the severity of the outcomes of this illness. Moreover, beat-to-beat blood pressure variability, which is also a valuable tool to investigate cardiovascular regulation, is poorly investigated in COVID-19 syndrome (4). On top of that, few studies have evaluated cardiovascular variability responses to challenging maneuvers such as the head-up tilt test (HUTT) in patients with PCS. Of note, Faria et al. (5) have already demonstrated that COVID-19 survivors exhibit reduced exercise capacity, while the neurovascular response to sympathoexcitatory challenges determined by the handgrip exercise was preserved (6). We hypothesize that patients with PCS will show changes in HR and AP variability at either basal conditions or during HUTT, when compared to control healthy subjects. Moreover, we expect that the lower HRV and higher APV indicating an autonomic dysfunction with sympathetic predominance in PCS patients, and the attenuated responses of HRV and AP variability indices during HUTT indicating a marked impairment of autonomic control, will return to normal after 6 months of follow up.

Furthermore, we highlight that the most commonly used HRV approach to evaluate autonomic function is the spectral analysis of the RR series. However, the symbolic analysis proposed by Guzzetti et al. (7) and used in the present study has been shown to be more sensitive to detecting cardiac autonomic changes at either clinical (8, 9) or experimental levels (10).

It is well known that the SARS-CoV-2 emerged in the city of Wuhan (China), causing the occurrence of unusual viral pneumonia, leading to the coronavirus disease 2019 (COVID-19) (11). Besides significant pulmonary damage, SARS-CoV-2 infection also leads to cardiocirculatory abnormalities, for instance, myocarditis, pericarditis, arrhythmia, heart failure, cardiogenic shock, and abnormalities of coagulation (12). It is well known that the infection with SARS-Cov-2 may lead to marked cardiac autonomic dysfunction (13). Soliński et al. (1) highlighted that probably due to the prolonged inflammatory process induced by the infection with SARS-CoV-2, autonomic dysfunction might persist long after viral shedding. Apropos, it is not really known what is behind the post-COVID condition. Central nervous system inflammation is one of the hypotheses of long-term complications in patients who have been infected with SARS-Cov-2 (14). Previous studies showed significant changes in HRV parameters in severe (including fatal) infections with SARS-CoV-2 (1). However, few studies have comprehensively examined the autonomic cardiovascular control in previously asymptomatic, or mildly symptomatic individuals exposed to SARS-Cov-2 (1). In this regard, the results obtained by Soliński et al. (1) suggested an increase in the parasympathetic function, contrasting with the results obtained by Stute et al. (2), who found an increase in sympathetic activity by measuring the muscle sympathetic nerve activity (MSNA) in young adults recovering from SARS-CoV-2 infection.

On the other hand, Barizien et al. (3) reported that increasing numbers of COVID-19 patients, continue to show symptoms months after recovering. Moreover, they have also expressed that autonomic dysfunction, which can aggregate all neurological symptoms, has yet to be prominently reported (3). Nevertheless, their study concluded that patients with long COVID-19 might exhibit dysautonomia characterized by marked changes in HRV indices.

Notable studies dealing with blood pressure variability and COVID-19 suggested that hypertension itself, and its target organ damage and complications, might be a high risk and the cause of fatality for patients with PCS (15, 16). In a retrospective analysis, Li et al. (17) investigated day-by-day blood pressure variability and its association with clinical outcomes (critical vs. severe and discharged) in hospitalized patients with COVID-19. Thus, these authors reached the conclusion that in patients with COVID-19 the blood pressure variability was exacerbated and associated with worse clinical outcomes (17). However, regardless of whether the assessed blood pressure variability is a risk indicator, it may serve as an important biological marker for clinical outcomes of COVID-19 (17). Nandadeva et al. (18) reported that neither ambulatory AP nor laboratory AP were different between control and COVID-19 patients; however, they found a significant inverse relationship with time since COVID-19 diagnosis was established, i.e., greater AP was related to more recent infection.

Until now, few reports have assessed the relationship between day-to-day blood pressure variability and mortality in COVID-19 (4, 16). In addition to strict blood pressure control, it might be important to minimize day-to-day blood pressure variability to reduce mortality in COVID-19 patients (19).

On the other hand, it should be emphasized that the literature is remarkably poor concerning the investigation of cardiovascular responses to challenges, such as the head-up tilt test (HUTT), in patients with PCS. It is quite curious that Eldokla and Ali (20) reported that in their case series, most patients with long-COVID presenting to their laboratory with orthostatic intolerance had no significant HUTT abnormalities; while only 3 patients met the criteria for Postural Orthostatic Tachycardia Syndrome (POTS). Despite this, Seeley et al. (21) showed that the prevalence of autonomic symptomatology for POTS was high in those patients with post-acute sequelae of COVID-19, leading to poor health-related quality of life. What is more, these authors Eldokla and Ali (20) reported that when they calculated the Composite Autonomic Severity Score (CASS) of patients with long-COVID presenting with orthostatic intolerance, which is a validated tool to access the quantification of the severity and distribution of autonomic dysfunction (22), they observed that these long-COVID patients had a low CASS, indicating mild or minimal autonomic dysfunction. Nevertheless, it is worth noting that Luck et al. (23) demonstrated that the most significant autonomic abnormality was the incapacity of three collegiate athletes, who tested positive for COVID-19, to complete a 10-min orthostatic challenge. However, Raj et al. (24) reported that while most people with COVID-19 illness recover completely, others continue to experience chronic and diverse symptoms, including autonomic manifestations. Besides, Stute et al. (2) observed that resting sympathetic activity, but not heart rate or blood pressure, may be elevated following SARS-CoV-2 infection. In addition, they stated that cardiovascular and perceptual responses to physiological stress in PCS might be altered.

Thus, the present study investigated the HRV and AP (arterial pressure) variability at rest and during the HUTT in patients with PCS who reported remnant cardiovascular symptoms, such as tachycardia and postural hypotension, persisting for at least three months. In addition, a group of patients with PCS was reassessed after 6.2 ± 1 months, i.e., from 159 to 224 days after the first evaluation. HRV and AP variability results suggest an autonomic dysfunction with sympathetic predominance in PCS patients. As well as this, the HRV and AP variability indices during HUTT indicate a marked impairment of autonomic control. Of note, similar to the findings from spectral analysis, the symbolic analysis – a reliable tool for the assessment of rapid changes of cardiac autonomic modulation induced by a graded HUTT (8) – showed that the occurrence of the percentage of 2UV (two unlike variation) pattern of RR (beat-to-beat interval) – a parameter that reflects changes in vagal modulation – decreased during the HUTT in control subjects, but not in patients with PCS. However, in the reassessed PCS patients, a decrease in the percentage of 2UV was also observed during the HUTT.

## Methods

### Participants

The study was performed among patients attending a post-COVID-19 outpatient clinic at the University Hospital from the Ribeirão Preto Medical School – University of São Paulo (HCFMRP/USP), Ribeirão Preto, SP, Brazil. Adult patients (*N* = 16) with PCR confirmed COVID-19 with symptom onset during February 1st – December 31st, 2021, of both sexes, aged between 32 and 61, were eligible for the study. All patients required hospitalization during the acute phase of COVID-19, and exhibited after recovering from COVID-19 residual cardiovascular symptoms, such as tachycardia and postural hypotension, i.e., patients with the PCS exhibited cardiovascular symptoms. This was a subsample of the RECOVIDA Project (25), a cohort study aiming to comprehensively describe the long-term symptoms observed among patients surviving the acute phase of COVID-19 and to investigate the physical, emotional, and social impact associated with them. Patients were consecutively recruited in an outpatient clinic named Post-COVID-19 Ambulatory. Most patients were referred to the clinic after being discharged from a hospital admission due to severe or critical COVID-19 (7, 25). A group of 10 out of the 16 patients with PCS were reassessed after approximately 6 months. To build a control group for this study, we selected exams (*N* = 22) that were stored in a database of the Division of Cardiology of the Department of Internal Medicine from the Ribeirão Preto Medical School – University of São Paulo and performed at the HCFMRP/USP, following the same protocol applied to the patients with PCS. These exams were performed before the COVID-19 pandemic in adult patients of both sexes, aged between 31 and 69 years old, opting for exams of healthy patients with fewer comorbidities and use of medications. The patients were submitted to the HUTT to evaluate for a possible neurocardiogenic syncope. These patients were considered robust after clinical investigation, displaying normal responses to the HUTT.

Control patients were randomly selected, with preference given to healthy individuals with fewer comorbidities, using medications with low influence on HRV, and age similar to the PCS patients. No race/ethnicity was taken into account when selecting the Control and PCS patients. Concerning the female participants of both groups (PCS and Control), the menopause factor and period of the menstrual cycle were not taken into account when performing the HUTT and selecting the exams in the database.

The data collection and the analysis protocols were conducted following The Code of Ethics of the World Medical Association (Declaration of Helsinki) and authorized by the Research Ethics Committee of HCFMRP/USP (CAAE:31172720.9.0000.5440).

### Data collection

Participants were recruited from a post-COVID-19 outpatient clinic at the University Hospital of the Ribeirão Preto Medical School – University of São Paulo, Ribeirão Preto, SP, Brazil.

The data collection was performed in a controlled temperature and humidity room, with emergency support, located at the Division of Cardiology of the HCFMRP/USP. All tests were performed on the same day to avoid circadian rhythm influences.

For participants who used drugs that affect the autonomic nervous system, such as β blockers, discontinuation of the medication was recommended 24 h before the examination.

Participants were positioned securely on a tilt test table and attached to electrocardiogram (ECG) electrodes (lead II) and a finger cuff for non-invasive AP monitoring (Finometer PRO, Finapres Medical Systems, Amsterdam, Netherlands). ECG signal was filtered (0.5–100 Hz), amplified (8811A, Hewlett Packard, Palo Alto, California, USA), and sampled (1 kHz) in an IBM/PC equipped with an analog-to-digital interface (DI720, DATAQ, Akron, Ohio, USA). The signal from the Finometer PRO system was sampled simultaneously with ECG. Patients were breathing spontaneously during data collection, without pacing.

The subjects were instructed to remain relaxed with minimal movement and no talking for 10 min for baseline (rest) ECG and AP recordings. Subsequently, the HUTT was performed by carefully angling the table to 70° and keeping it in this position for the next 10 min while ECG and AP were continuously recorded. Finally, the table was returned to rest, where the subjects were allowed to recover.

Participants’ recruitment and data collection covered February 1st, 2021, and June 31st, 2022.

### Data processing and analysis

ECG and AP recordings were processed using the computer software LabChart (ADInstruments, Dunedin, New Zealand), capable of generating beat-to-beat time series with values of RR intervals as well as systolic, diastolic, or mean AP. Series of successive values of RR intervals and systolic AP were generated for the basal period and during HUTT.

Spurious values from recording artifacts or ectopic beats were removed from the time series using the following procedure: a moving average window of 10 to 50 values was used to calculate the series baseline. Next, upper and lower thresholds were defined as the baseline shifted up and down by a rate of 0.1 to 0.2 times the mean. All PI (Pulse Interval) values above the upper or below the lower threshold were replaced using linear interpolation. The study did not use a series in which removals exceeded 1% of the total number of PI values.

Linear and non-linear indices of HRV and systolic AP variability were calculated using the customized freely available computer software PyBios (26) as follows:

*Time domain:* Standard deviation of successive cardiac intervals (SDNN) and root mean square of the successive differences (RMSSD) from beat-to-beat RR intervals were calculated. The standard deviation of systolic AP values was calculated as an index of overall AP variability.

*Frequency domain (spectral analysis):* Both RR intervals and systolic AP time series were resampled at 4 Hz using cubic spline interpolation and divided into half-overlapping segments of 512 points. After Hanning windowing, each segment had its spectrum calculated using the Fast Fourier Transform, and the spectra of RR intervals were integrated into low- (LF: 0.04–0.15 Hz) and high-frequency (HF: 0.15–0.5 Hz) bands. In contrast, the spectra of systolic AP were integrated only at LF. The median of the LF and HF powers from all segments were considered for each patient and expressed in absolute or normalized units.

*Symbolic analysis:* Non-linear analysis of symbolic dynamics (8, 27) was performed as follows. Series of RR intervals (or systolic AP values) were split into segments of 500 values overlapped by half. For each segment, the full range of values was divided into six uniform distributed levels, and symbols were assigned to each value according to the level it belonged to. Sequences of three consecutive symbols were analyzed and classified into one of the four following families according to their variation pattern: zero variation (0 V), where symbols are all the same; one variation (1 V), where two consecutive symbols are equal, and the remaining is different; two like variations (2LV), where variations between symbols are in the same direction, creating an ascending or descending ramp; and two unlike variations (2UV), where the variations between symbols are opposite, forming a peak or valley. The percentage of occurrence of each family was computed for each segment, and the median values of the segments were taken to represent the whole series of RR intervals (or systolic PA).

*Sample entropy (SampEn):* SampEn measures the irregularity of the time series, where the higher the SampEn, the more irregular (unpredictable) the time series is. SampEn parameters were set to *m* = 2 and *r* = 15% of time series standard deviation, where *m* is the length of the sequences (number of RR intervals) considered to calculate the predictability of the time series and *r* is the tolerance factor (28).

### Statistical analysis

Data are presented as mean ± SEM. The *Kolmogorov–Smirnov* test was applied to test the normality of data distribution. Two-way analysis of variance (2-way ANOVA) was used to compare groups and HUTT when the data set was normally distributed; otherwise, an analysis of variance on ranks (Kruskal-Wallis test) was applied, whenever the data set deviated from the normal population. When differences were found, the data were compared by the *post hoc* parametric *Tukey* or non-parametric *Mann–Whitney U*-test depending on the proximity to the normal population of the data set. Intragroup comparisons, before and during HUTT, were performed by paired *Student t*-test or *Wilcoxon* rank test when appropriate. The significance level was set at *p* < 0.05.

## Results

### Description of the study population sample

A total of 22 individuals were evaluated as Control, and 16 patients were the PCS group. In addition, 10 out of 16 post-COVID-19 syndrome (PCS) patients were reassessed approximately 6 months later. The demographic and clinical characteristics of the participants are described on Table 1, while the echocardiographic characteristics are described on Table 2. Patients with PCS and the reassessed PCS patients showed significant tachycardia compared to the Control individuals. The echocardiographic analysis did not show any significant differences among all groups studied.

**Table 1:** Demographic and clinical characteristics of post-COVID-19 syndrome (PCS) patients, Reassessed PCS patients, and their matched Control.

Parameters	Control (*N* = 22)	PCS patients (*N* = 16)	Reassessed PCS patients (*N* = 10)
Age (Years)	52 [31–69]	45 [32–61]	46 [32–61]
Sex	F (68%) M (32%)	F (56%) M (44%)	F (60%) M (40%)
Weight (kg)	77 [56–135]	85 [55–95]	82 [55–95]
SBP (mmHg)	124 ± 3	123 ± 4	125 ± 4
DBP (mmHg)	82 ± 2	78 ± 3	76 ± 4
Heart Rate (bpm)	74 ± 4	99 ± 3*	103 ± 3*
AH	No (68%) Yes (32%)	No (63%) Yes (37%)	No (40%) Yes (60%)
Use of NSAIDs	No (91%) Yes (9%)	No (94%) Yes (6%)	No (90%) Yes (10%)
Previous use of β Blockers	No (82%) Yes (18%)	No (81%) Yes (19%)	No (60%) Yes (40%)
Previous use of Anti-Hypertensive Drugs	No (68%) Yes (32%)	No (63%) Yes (37%)	No (40%) Yes (60%)

Age and weight are expressed as median [interquartile range]; Sex, AH (Previous Diagnosis of Hypertension), NSAIDs, Previous Use of β Blockers, and Previous Use of Anti-Hypertensive Drugs are expressed as %; SBP (Systolic Blood Pressure), DBP (Diastolic Blood Pressure), and Heart Rate are expressed as mean ± SEM. *p < 0.001, versus CONTROL were considered significant from the one-way ANOVA.

**Table 2:** Echocardiographic characteristics of post-COVID-19 syndrome (PCS) patients, Reassessed PCS patients and their matched Control.

Parameters	Control (*N* = 13)	PCS patients (*N* = 15)	Reassessed PCS patients (*N* = 09)
LAVI (mL/m^2^)	32.7 ± 4.1	25.8 ± 2.8	29.0 ± 4.3
LVEDD (mm)	47.5 ± 2.0	46.2 ± 2.2	45.5 ± 3.6
LVEF (%)	63.5 ± 2.3	57.4 ± 2.4	57.4 ± 2.7

Values are expressed as mean ± SEM. LVEDD, ventricular end-diastolic diameter; LVEF, left ventricular ejection fraction; LAVI, left atrial volume index. No significant differences were found using one-way ANOVA.

### Heart rate variability indices at rest: first recording and reassessment

At their first assessment, patients with PCS showed higher basal HR than the control subjects (84 ± 4 vs. 71 ± 3 bpm, *p* = 0.004). The patients reassessed after 6 months remained with higher basal HR (79 ± 3 bpm vs. 87 ± 4 bpm, *p* = 0.002, at first recording and reassessment, respectively).

**Figure 1:**
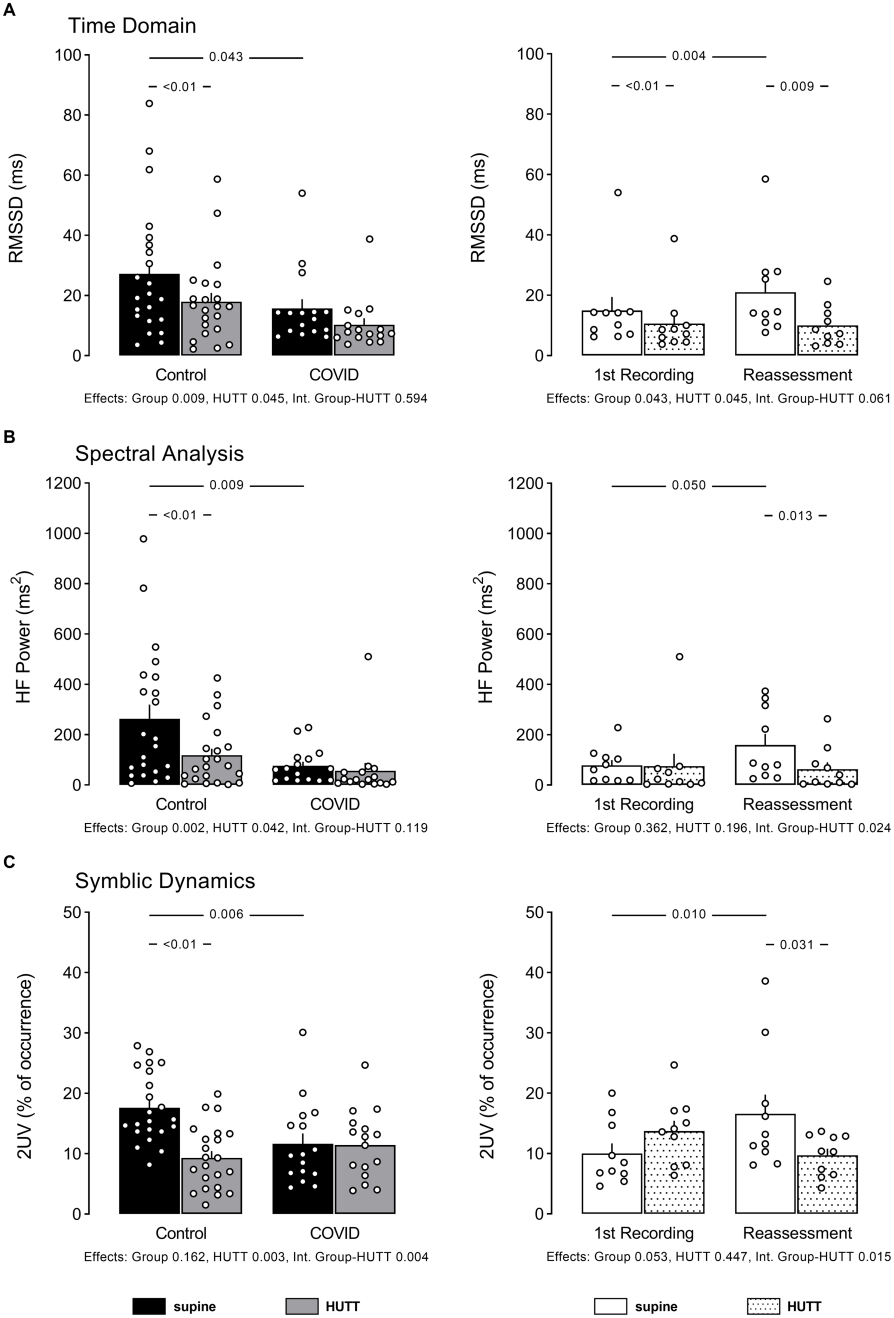
Individual data and mean ± EPM values of RR interval variability indices linked to vagal modulation of the heart at supine and head up tilt. **(A)** Shows root mean square of successive differences squared of RR intervals (time domain), **(B)** shows high-frequency power of RR intervals spectra (spectral analysis), and **(C)** shows percentage of occurrence of sequences with 2 variations according symbolic analysis as described by Porta et al. Left column brings the comparison between control (*N* = 22) and patients recovered from COVID-19 (*N* = 16). Right column brings the comparison between patients recovered from COVID-19 at their 1st recording and reassessment after 6 months (*N* = 10). Numbers between bars show *p* value obtained by Tukey or Mann Whitney U test, accordingly. Numbers below bar graphs show *p* values obtained by 2-Way ANOVA for effect of group (control vs. COVID), effect of HUTT (supine vs. orthostatic position) and interaction group-HUTT.

**Figure 2:**
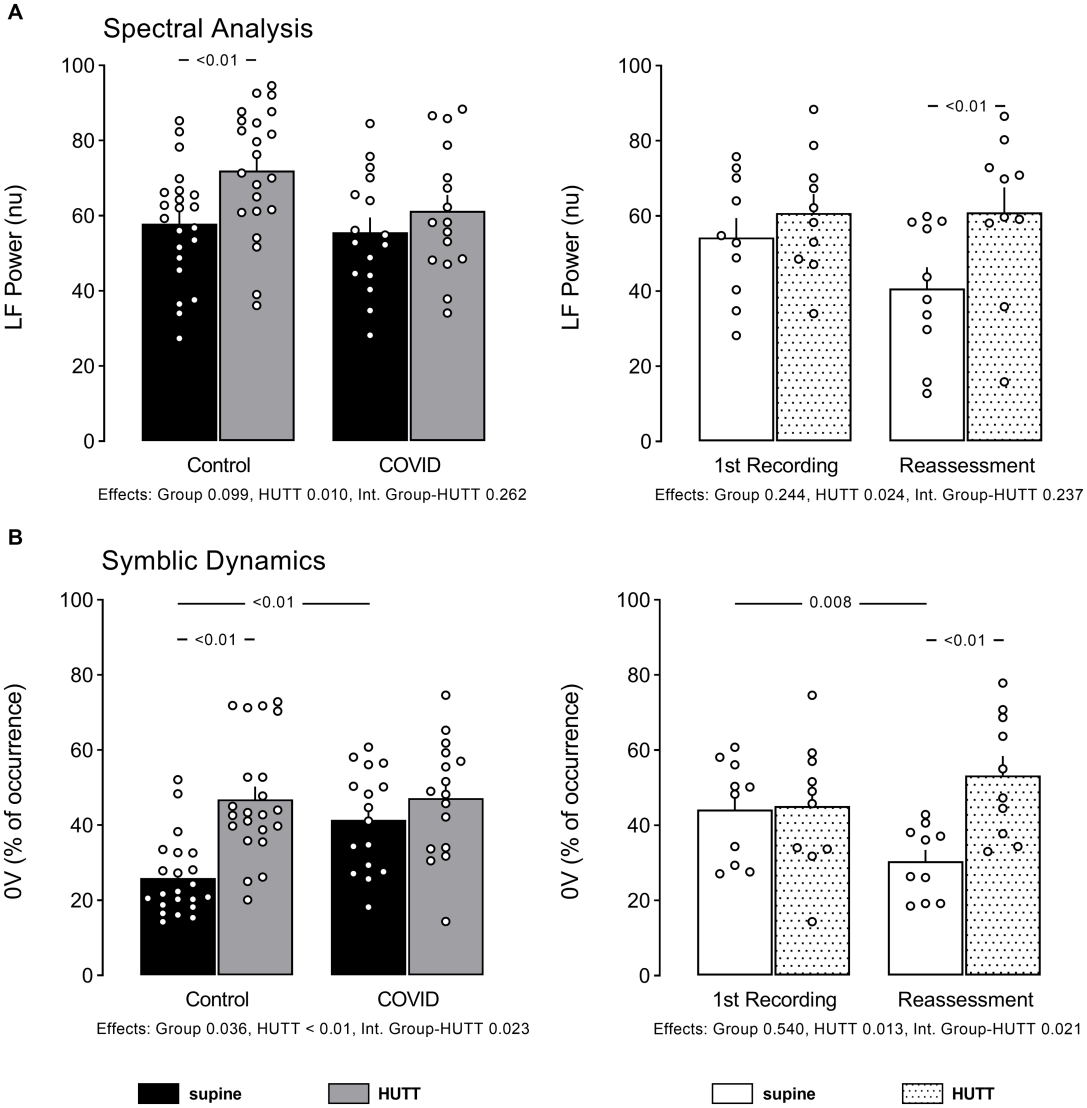
Individual data and mean ± EPM values of RR interval variability indices linked to sympathetic modulation of the heart at supine and head up tilt. **(A)** Shows high-frequency power of RR intervals spectra (spectral analysis) and **(B)** shows percentage of occurrence of sequences with no variation according symbolic analysis as described by Porta et al. Left column brings the comparison between control (*N* = 22) and patients recovered from COVID-19 (*N* = 16). Right column brings the comparison between patients recovered from COVID-19 at their 1st recording and reassessment after 6 months (*N* = 10). Numbers below bar graphs show *p* values obtained by 2-Way ANOVA for effect of group (control vs. COVID), effect of HUTT (supine vs. orthostatic position) and interaction group-HUTT.

**Figure 3:**
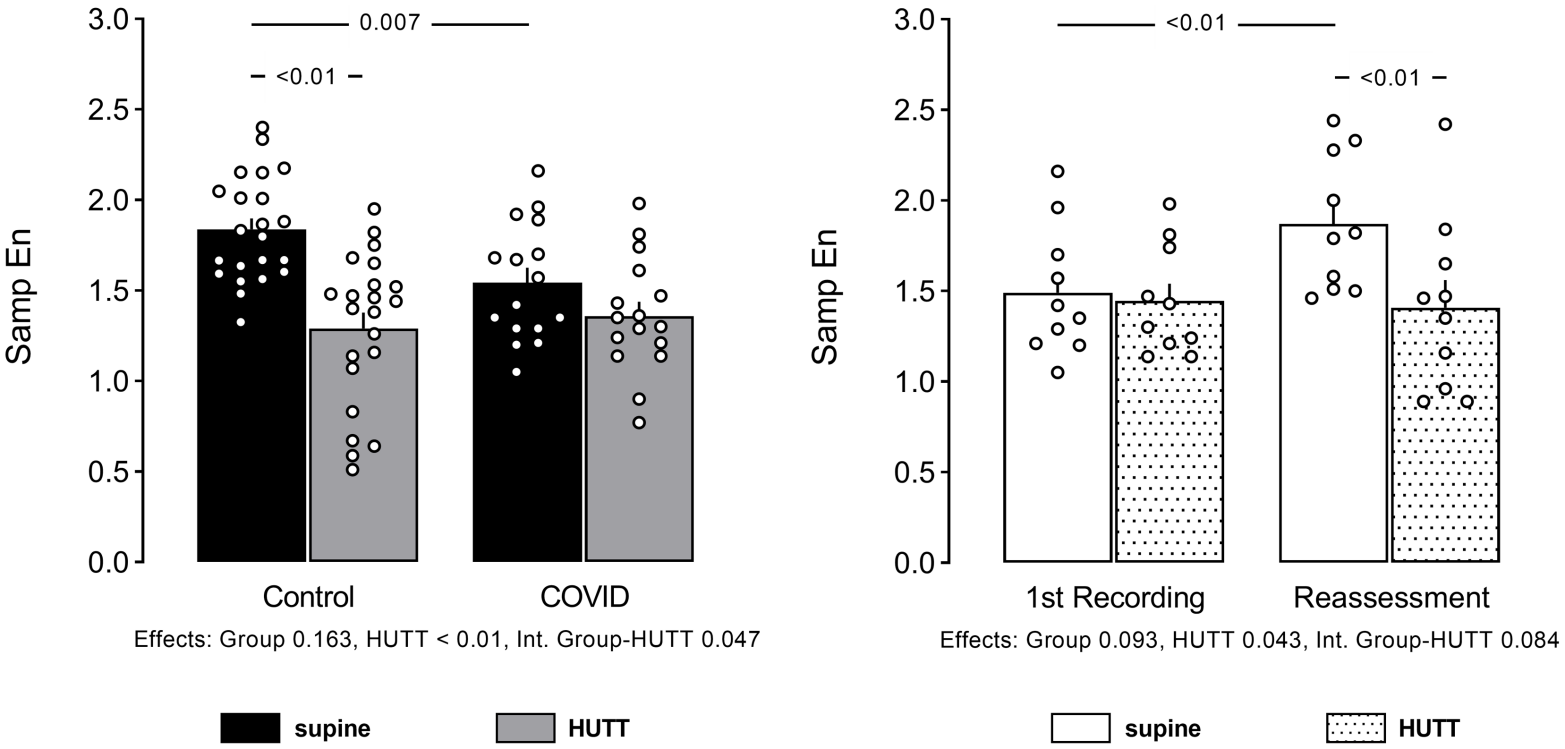
Individual data and mean ± EPM values of Sample Entropy (SampEn) of RR interval series at supine and head up tilt. Left column brings the comparison between control (*N* = 22) and patients recovered from COVID-19 (*N* = 16). Right column brings the comparison between patients recovered from COVID-19 at their 1st recording and reassessment after 6 months (*N* = 10). Numbers below bar graphs show *p* values obtained by 2-Way ANOVA for effect of group (control vs. COVID), effect of HUTT (supine vs. orthostatic position) and interaction group HUTT.

The data of HRV examined in the time and frequency domain (spectral analysis), and through non-linear analysis (8, 10) are displayed in Figures 1, 2. Values of Sample Entropy from the RR series of the two groups studied are shown in Figure 3.

Patients with PCS showed lower RMSSD, remarkably lower power of RR spectra at HF, and lower occurrence of 2UV patterns from the symbolic analysis, compared to their control counterparts. Of note, the patients reassessed 6 months later recovered the HF power of RR spectra and occurrences of 2UV pattern of symbols, but not the RMSSD.

The power of RR spectra at the LF band was not different between the two groups, contrasting with the higher incidence of 0 V pattern from the symbolic analysis shown by patients with PCS. At reassessment, the LF power of RR spectra remained similar to the first evaluation, contrasting with the occurrence of 0 V pattern of symbols that decreased with time.

Sample entropy was smaller in patients with PCS at their first assessment. However, SampEn from PCS patients reassessed after 6 months was back to normal, compared to their first recording.

### Heart rate variability indices during the head-up tilt test: first recording and reassessment

HUTT elicited a tachycardia response in both groups, i.e., control subjects (71 ± 3 to 80 ± 4 bpm, *p* < 0.001) and patients with PCS (84 ± 3 to 94 ± 3 bpm, *p* < 0.001). At reassessment, patients with PCS also displayed significantly increased HR during the HUTT (79 ± 3 vs. 89 ± 4 bpm, *p* < 0.010, at first recording and reassessment, respectively).

In addition to showing baseline (rest) values, Figures 1, 3 also show HRV indices in response to HUTT in control subjects and patients with PCS.

As expected, the results in the time domain demonstrated a reduction of the RMSSD during the HUTT in both, control subjects and patients with PCS, even though patients with PCS showed a smaller change of RMSSD elicited by the HUTT than their control counterparts. However, the HUTT applied to the patients with PCS reassessed after 6 months, determined a RMSSD response similar to that observed during the first recording.

Looking at the spectral analysis of HRV, the power of RR spectra at HF was, as expected, markedly reduced by HUTT in control subjects. However, HUTT did not affect the HF power of RR spectra in patients with PCS. Nevertheless, in patients reassessed after 6 months, the HF power of RR spectra was reduced by HUTT.

The power of RR spectra at the LF band was increased by HUTT only in control subjects, and not in patients with PCS at their first evaluation. However, when reassessed after 6 months, HUTT elicited a significant increase in LF power of RR spectra in patients with PCS.

Similar to the findings from spectral analysis, the symbolic analysis showed that the occurrence of 2UV decreased with HUTT in control subjects, but not in patients with PCS. Nevertheless, in the reassessed patients, HUTT decreased the percentage of 2UV.

Also, similarly to the LF power of RR spectra, the occurrence of patterns with 0 V increased with HUTT only in the control subjects at first recording; but, in the reassessed patients, HUTT elicited an increase in this index.

In line with the other findings, SampEn decreased with HUTT only in control subjects at their first evaluation. However, HUTT significantly reduced SampEn in patients with PCS during their reassessment.

### Arterial pressure and its variability at rest: first recording and reassessment

No significant difference was found concerning the systolic AP at rest between control subjects and patients with PCS (129 ± 3 vs. 137 ± 3 mmHg, *p* = 0.079). Similarly, the reassessed patients also showed similar systolic AP values (141 ± 5 vs. 131 ± 5 mmHg, *p* = 0,052, at first recording and after 6 months, respectively).

**Figure 4:**
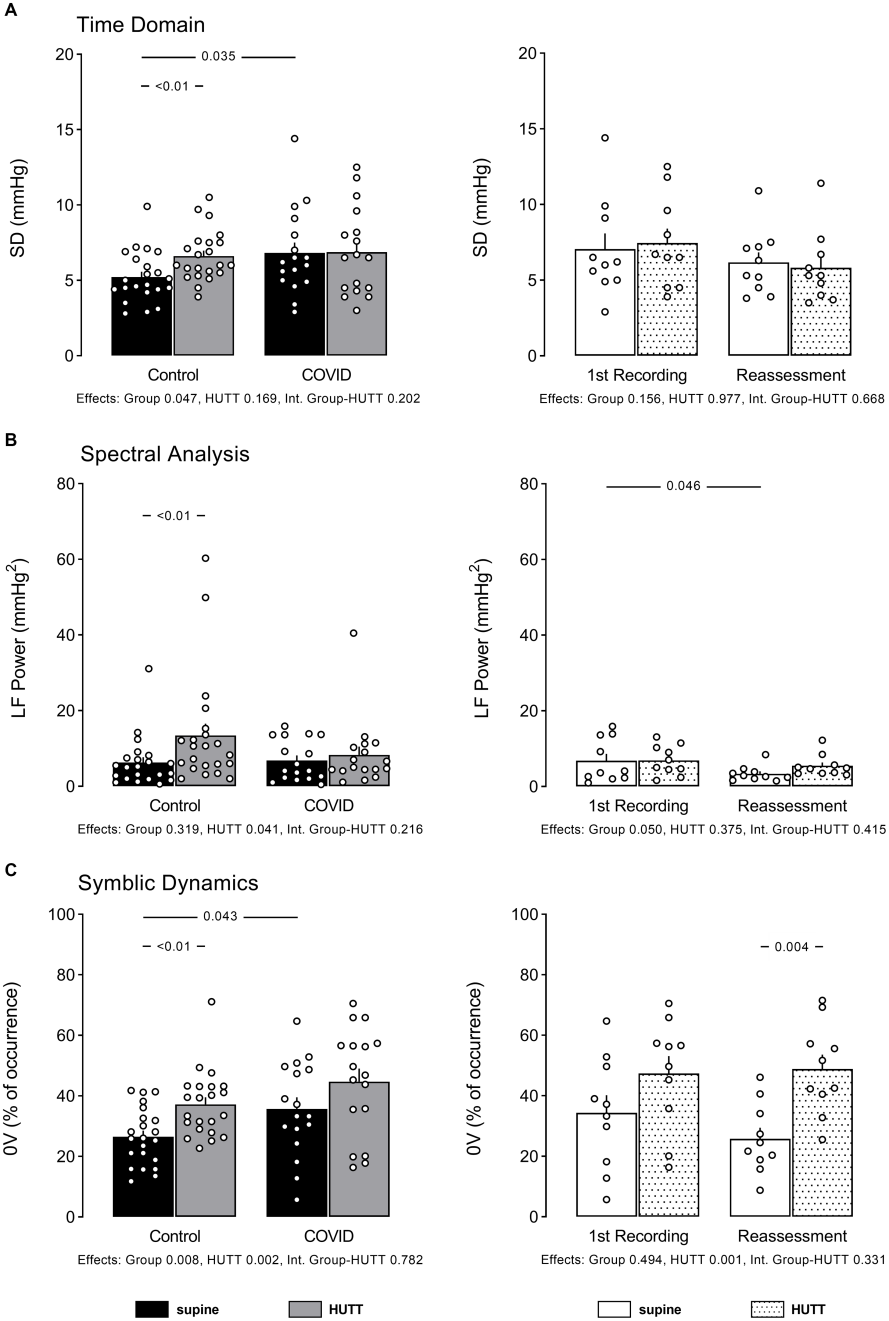
Individual data and mean ± EPM values of systolic arterial pressure variability indices at supine and head up tilt. **(A)** Shows standard deviation of pressure values (time domain), **(B)** shows low frequency power of systolic pressure spectra (spectral analysis), and **(C)** shows percentage of occurrence of sequences with no variations according symbolic analysis as described by Porta et al. Left column brings the comparison between control (*N* = 22) and patients recovered from COVID-19 (*N* = 16). Right column brings the comparison between patients recovered from COVID-19 at their 1st recording and reassessment after 6 months (*N* = 10). Numbers between bars show *p* value obtained by Tukey or Mann Whitney *U* test, accordingly. Numbers below bar graphs show *p* values obtained by 2-Way ANOVA for effect of group (control vs. COVID), effect of HUTT (supine vs. orthostatic position) and interaction group-HUTT.

The data of systolic AP variability examined in the time and frequency domain, as well as by the non-linear symbolic dynamics approach, are displayed in Figure 4.

Patients with PCS exhibited significantly higher overall pressure variability, as shown by the high SD of systolic AP at rest, compared to their control counterparts. The systolic pressure variability examined by spectral analysis showed similar values of LF power of pressure spectra in both groups studied. When the systolic AP variability was examined by symbolic analysis, a high occurrence of the pattern 0 V was observed in patients with PCS.

The patients reassessed after 6 months showed similar SD of systolic AP, similar LF power of AP spectra, and lower occurrence of 0 V compared to their first assessment.

### Arterial pressure variability responses to head-up tilt test: first recording and reassessment

HUTT did not change systolic AP in either control subjects (129 ± 3 vs. 131 ± 3 mmHg, *p* = 0.363) or patients with PCS (137 ± 3 vs. 138 ± 3 mmHg, *p* = 0.430).

Figure 4 also shows systolic AP variability indices in response to HUTT in control subjects and patients with PCS.

While the control subjects displayed increased systolic pressure SD during the HUTT, the patients with PCS did not exhibit any pressure response with this maneuver. At reassessment, no change of systolic pressure SD was observed between groups in response to HUTT.

As expected, HUTT elicited a marked increase in the LF power of pressure spectra in control subjects, while patients with PCS did not show any change in this parameter during HUTT. Nevertheless, in the reassessed patients, the LF power of AP spectra increased with HUTT.

Moreover, while the control individuals exhibited an increase of 0 V% during the HUTT, the patients with PCS showed no change of this parameter elicited by this maneuver. However, in both the first assessment and 6 months after, patients with PCS showed a normal response concerning 0 V% during the HUTT.

## Discussion

The present study investigated HR and AP variability in patients with PCS with symptoms of cardiovascular disorders compared to control subjects evaluated before the COVID-19 pandemic. In addition, 10 out of 16 patients with PCS were reassessed after six months. Besides the cardiovascular variability at rest, the subjects of the present study were also evaluated during HUTT, a challenge to the cardiovascular system.

### Impairment of parasympathetic function

The analysis of HRV of patients with PCS at rest demonstrated an attenuation of the RMSSD and HF power of RR spectra, combined with a significant reduction of the occurrence of the 2UV pattern from the symbolic analysis. Therefore, these findings suggest that patients with PCS exhibit reduced vagal – parasympathetic – modulation of the heart at rest. Apropos, the patients with PCS on their first recording, as expected, showed higher basal HR compared to control subjects.

These findings align with previous observations from the literature (29, 30), suggesting that long COVID-19 has led to an impairment of parasympathetic function. Furthermore, it was also observed in the current study lower SampEn of RR Intervals in the patients with PCS. This observation also aligns with those published by Aliani et al. (31), who showed a decrease in entropy related to the severity of COVID-19. Aranyó et al. (30) stated that the imbalance of the cardiac autonomic modulation might explain, for instance, the inappropriate sinus tachycardia (IST) in patients with PCS, an outcome also observed in some patients from the current study exhibiting PCS.

### Imbalance of the autonomic nervous system

Moreover, Leitzke et al. (32) have pointed out that patients with an increased risk of a more severe COVID-19 showed a disturbed balance of the autonomic nervous system, particularly with impairment of vagal function (33). In contrast, Latchman et al. (34) observed no difference in parasympathetic modulation, sympathetic modulation, and BRS between young adults who had COVID-19 versus young adults who never had COVID-19. These findings suggest a preserved autonomic nervous system function and baroreflex sensitivity in young adults after COVID-19. Additionally, looking at identifying a cut-off point value for HRV associated with elevated risk across a range of known risk factors, Leitzke et al. (32) provided the first evidence that changes in RMSSD may be related to high risk across a range of established cardiovascular risk factors.

However, the findings from the current study contrast with the observations from other studies in patients with a history of COVID-19, who found an increased RMSSD (13, 35) consistent with a parasympathetic overactivation (1). Asarcikli et al. (13) have also found an LF/HF ratio increase, which is compatible with sympathetic overactivity. On the other hand, when Salem et al. (36) investigated the post-acute effect of SARSCoV-2 infection on cardiovascular autonomic activity in patients with PCS, who were exposed to the infection at least three months before, they concluded that despite several parameters of HRV being numerically reduced in these patients with PCS, they were not statistically significant.

### Sympathetic modulation of the heart was not altered at rest

It was also observed in the current study that the patients with PCS exhibited an LF power of HRV, indicating that the sympathetic modulation of the heart was not altered at rest; however, the increase in the occurrence of 0 V contradicts this observation, indicating an increased sympathetic modulation of the heart in these patients. Notably, Asarcikli et al. (13) found an LF/HF ratio increase, which is also consistent with sympathetic overactivity. Finally, Stute et al. (2) observed a rise in resting sympathetic activity in young adults who tested positive for SARS-CoV-2 when measuring muscle sympathetic nerve activity (MSNA). However, Skow et al. (37) suggested the incidence of a transient impact of COVID-19 on cardiac autonomic function, which appears mild and unrelated to persistent symptoms in young adults. Furthermore, Stute et al. (38) have demonstrated that sympathetic activation prior to sympathoexcitatory challenges determined by the HUTT indicate that resting sympathetic activity, evaluated through the MSNA may be reduced during the recovery from SARS-CoV-infection.

### Analysis of arterial pressure variability

The analysis of AP variability showed higher overall pressure variability, confirmed by the higher values of SD of systolic AP in patients with PCS. In addition, these patients also showed higher LF Power of AP spectra and higher occurrence of 0 V patterns compared to their control counterparts. These findings strongly indicate an overactivity of vascular sympathetic modulation at rest in these patients (8, 39–41).

It is worthy of note that the literature displays few studies of blood pressure variability in COVID-19. Nevertheless, an autonomic nervous system imbalance has been suggested to determine the severity of COVID-19 (32, 42). Moreover, the data from the study of He et al. (43) provided an essential contribution to this notion; they are considered within the context of the precise pathophysiology underlying the relationship between COVID-19 infection and day-to-day BP variability (19). In line with this understanding, Li et al. (17) observed more significant systolic arterial pressure variability in critically ill patients when compared with their severe and discharged counterparts. This conclusion came from investigating day-by-day blood pressure variability and its association with clinical outcomes (critical vs. severe and discharged) in hospitalized patients with COVID-19 (17).

### Head-up tilt test and COVID-19

It should be emphasized that the literature is remarkably poor concerning investigating challenging maneuvers to the cardiovascular system, such as the HUTT with COVID-19. It is quite curious that Eldokla and Ali (20) reported that most patients presenting long-COVID in their laboratory with orthostatic intolerance had no significant HUTT abnormalities. Only three patients met the criteria for Postural Orthostatic Tachycardia Syndrome (POTS).

It is well known that passive HUTT promotes graded changes in the sympathovagal balance (44). Thus, the HUTT was used in the current study to characterize the derangement of the sympathovagal balance response of the heart and blood vessels in patients with PCS.

As expected, the time domain results demonstrated a significant reduction of the RMSSD during the HUTT in both control subjects and patients with PCS. In contrast, patients with PCS displayed a noticeable attenuation of this response compared to their control counterparts. As well as this, an expected decrease was also observed concerning the HF power of RR interval spectra and a reduction of the 2UV pattern in control individuals but not in patients with PCS. Likewise, an expected increase of the LF power of RR intervals was observed with an increase in the occurrence of 0 V pattern in the control subjects but not in patients with PCS during HUTT. These findings indicate a noxious effect of SARS-CoV-2, which probably affected the response of the sympathovagal balance elicited by HUTT in patients with PCS.

SampEn is a non-linear index of heart rate dynamics, which describes the complexity and unpredictability of RR interval behavior. It is linked to the vulnerability of the development of detrimental conditions such as atrial fibrillation and/or life-threatening ventricular arrhythmias (45). Likewise, other non-linear indices of HRV, such as the role of the autonomic modulation of the heart in the genesis of HRV entropy, are not defined. Nevertheless, when Silva et al. (46) examined the SampEn at multiple time scales with pharmacological blockade of cardiac autonomic receptors in rats, they found that entropy at short scales reflects vagal modulation of HR. In contrast, it would be associated with both sympathetic and parasympathetic cardiac modulation at a long time scales (46). Of note, another study in the literature corroborates this interpretation (47).

In line with the other findings of the present study, SampEn was lower in patients with PCS compared to control subjects and was not reduced during HUTT in these patients. Therefore, the results of SampEn strongly suggest a derangement in cardiovascular modulation in patients with PCS with cardiovascular symptoms.

Taking into account that the LF power of pressure spectra, as well as the occurrence of 0 V from symbolic analysis, did not increase during HUTT in patients with PCS, as it did in control subjects, this strongly suggests that the noxious effect of SARS-CoV-2 on cardiac control also affects the modulation of vascular smooth muscle in patients with PCS.

### HRV and sympathovagal modulation during the reassessment period

The results of HRV regarding the sympathovagal modulation of the heart from patients with PCS at the reassessment period, i.e., six months after the first recording, demonstrated that the RMSSD did not return to the values shown by the control subjects, contrasting with the data obtained in the frequency domain as well as the symbolic analysis. These last indices indicate that the imbalance of the sympathovagal cardiac modulation was normalized over six months.

However, these findings contrast with the observations from other studies (13, 29, 30, 35), which indicated that long COVID-19 exhibits an attenuation of the parasympathetic function.

Moreover, it was observed in the current study that the LF Power of HRV was similar in patients with PCS and control subjects at either the first recording or reassessment. These findings contrast with 0 V – symbolic analysis – which was exacerbated during the first recording but returned to normal during the reassessment period. Together, these findings indicate a normal sympathetic modulation of the heart within six months in patients with PCS. These findings align with the observations from Salem et al. (36), who investigated the post-acute effect of SARSCoV-2 infection on cardiovascular autonomic activity, reactivity, and sensitivity, in patients who had the infection at least three months before. These authors observed that these patients displayed several parameters of HRV without significant changes.

It was also detected in the current study that the patients with PCS exhibited an increased occurrence of 0 V – symbolic analysis – at the 1st recording, which is coherent with the notion of an increased sympathetic modulation of the heart in these patients. Of note, this parameter was back to normal by the reassessment period, indicating a recovery of the sympathetic modulation of the heart under the circumstances.

### AP variability and sympathovagal modulation during the reassessment period

The AP variability from patients with PCS demonstrated that the SD and the occurrence of 0 V patterns of systolic AP – symbolic analysis – recovered to normal levels by the reassessment period. These findings indicate that the sympathetic modulation of the blood vessels in patients with PCS was back to normal when the three indices, i.e., SD (Time Domain), LF Power of AP spectra (Spectral Analysis), and the occurrence of 0 V patterns – Symbolic Analysis – were taken into account at the time of reassessment.

Thus, the results from the current study suggest a recovery of the sympathetic modulation of the vessels after six months.

The PSC patients exhibited a normal response, i.e., similar to their Control counterparts during the HUTT when reassessed after six months. Moreover, when the LF Power and the 0 V% were considered, the patients with PSC exhibited a similar response when reassessed after six months compared to their control counterparts. These data not only indicate that during the HUTT, there has been an increase in the sympathetic modulation of the blood vessels, but they also indicate that the sympathovagal balance of the vessels was back to normal within a 6-month time frame.

## Conclusion

In conclusion, the changes found in the HR and BP variability indices in patients with PCS suggest an autonomic dysfunction, with sympathetic predominance, in these individuals. The marked impairment of the autonomic control of the heart and vessels could lead to a higher risk of life-threatening cardiovascular events. However, one of the major findings of the present study was that the patients with PCS, who underwent the reassessment of the parameters studied, demonstrated that the noxious effect of the Post-COVID Condition related to these findings tends to fade away over time.

## Limitation of the current study

Not all PCS patients submitted to the First Recording returned for the Reassessment investigation, i.e., 10 out of 16 PCS patients returned for the Reassessment investigation.

The current study exclusively assessed the variability of systolic AP. Nonetheless, compelling evidence suggests that the variability in diastolic pressure values imparts complementary insights into vascular autonomic regulation, both in physiological and pathological contexts (48). Additionally, it is pertinent to acknowledge that modulations driven by shifts in central volume distribution, akin to those observed in the HUTT, impact the fluctuation of pulse pressure values.

## Data availability statement

The datasets presented in this study can be found in online repositories. The names of the repository/repositories and accession number(s) can be found at: The data sets gathered and/or analyzed during the current study are available from the corresponding author on reasonable request.

## Ethics statement

The studies involving humans were approved by Research Ethics Committee of HCFMRP/USP (CAAE:31172720.9.0000.5440). The studies were conducted in accordance with the local legislation and institutional requirements. The participants provided their written informed consent to participate in this study.

## Author contributions

HS contributed to the conception, supervision, and writing the manuscript. FS, MD, and AS contributed to the data collection and editing of the manuscript. LB and JC contributed to data collection. FB-R contributed to data collection, editing, and revising the manuscript. LJ and DM contributed to revising the manuscript for intellectual content. TB contributed to editing and revising the manuscript. RF contributed to data analysis and editing the manuscript. All authors read and approved the final manuscript.
